# Supplementary material for: Ultra-fast photodetectors based on high-mobility indium gallium antimonide nanowires
Source: Nat Commun. 2019 Apr 10;10:1664. doi: 10.1038/s41467-019-09606-y (PMC6458123; doi:10.1038/s41467-019-09606-y)
Supplement: Supplementary file 1 — Supplementary Information [file 41467_2019_9606_MOESM1_ESM.pdf]

Ultra-Fast Photodetectors based on High-Mobility Indium Gallium Antimonide Nanowires

D. Li, et al.

Mass Ratio of InSb:GaSb	Mass of Source (g)	Loss of Source (g)	Source Temperature (°C)	Substrate Temperature (°C)	Time (min)	Process Pressure (Torr)
10:1	1.0	0.17	710	540	60	3.1
20:1	1.0	0.26	720	520	120	1.9
30:1	1.0	0.31	740	505	180	2.1
40:1	1.0	0.32	750	510	180	2.1

Supplementary Table 1. The growth parameter employed for the synthesis of $In_xGa_{1-x}Sb$ nanowires. These growth parameters are optimized based on the nanowire surface morphology, geometry and electrical characteristics when configured into field-effect transistors.

Weight ratio of InSb:GaSb powder source	Nanowire Stoichiometry	Standard Deviation
10:1	$In_{0.15}Ga_{0.85}Sb$	In%=15±2%
20:1	$In_{0.22}Ga_{0.78}Sb$	In%=22±2%
30:1	$In_{0.09}Ga_{0.91}Sb$	In%=9±2%
40:1	$In_{0.28}Ga_{0.72}Sb$	In%=28±3%

Supplementary Table 2. The average nanowire composition determined from energy dispersive X-ray spectroscopy measurements. All associated values are extracted from more than 10 individual nanowires (NWs) for each sample group. It is noted that the powder source ratio of 30:1 would result in a relatively lower In concentration in $In_xGa_{1-x}Sb$ NWs. This inconsistency should not be an accidental result as we have repeated the experiments for many times and obtained the similar results. For the optimal growth condition of InGaSb NWs with around 9 % In content, the growth temperature is optimized at 505 ℃, which is the lowest temperature required among all NWs with different In content. We have also tried the higher growth temperature (>505 ℃) with the same powder mixing ratio, but this combination of growth parameters is out of the optimized process window grown with defective NWs with lots of surface coating (Supplementary Figure 2d). Since the growth temperature can drastically affect the chemical ratio of between Au and In in the AuIn alloy catalyst particle, which can subsequently affect the chemical composition of the grown NWs, we believe that the different growth temperature employed in this particular condition would contribute to the inconsistency here, where the powder source ratio of 30:1 (the third highest InSb case) yields the lowest In concentration (9%).

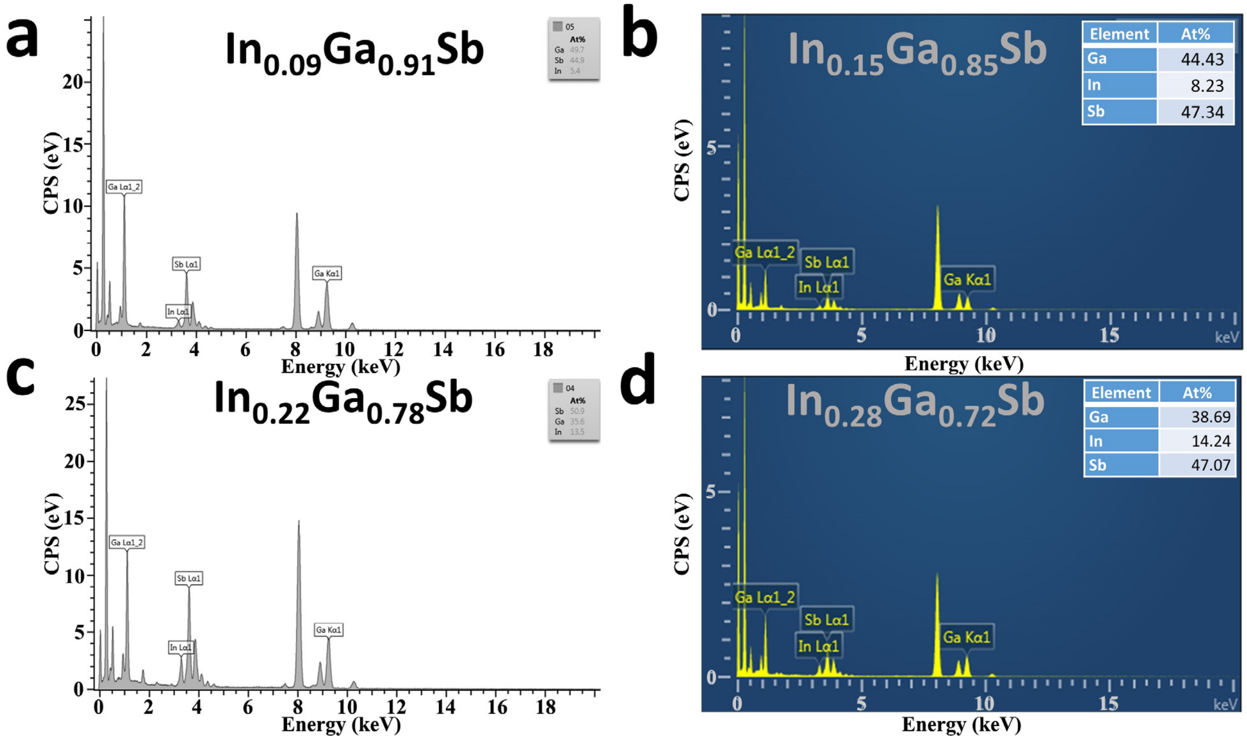

Supplementary Figure 1. Chemical composition of the obtained nanowires. Energy dispersive X-ray spectroscopy spectra of the as-grown $In_xGa_{1-x}Sb$ nanowires with different mixture ratios of InSb: GaSb (by weight) as the precursor source. (a) 30:1, (b) 10:1, (c) 20:1 and (d) 40:1.

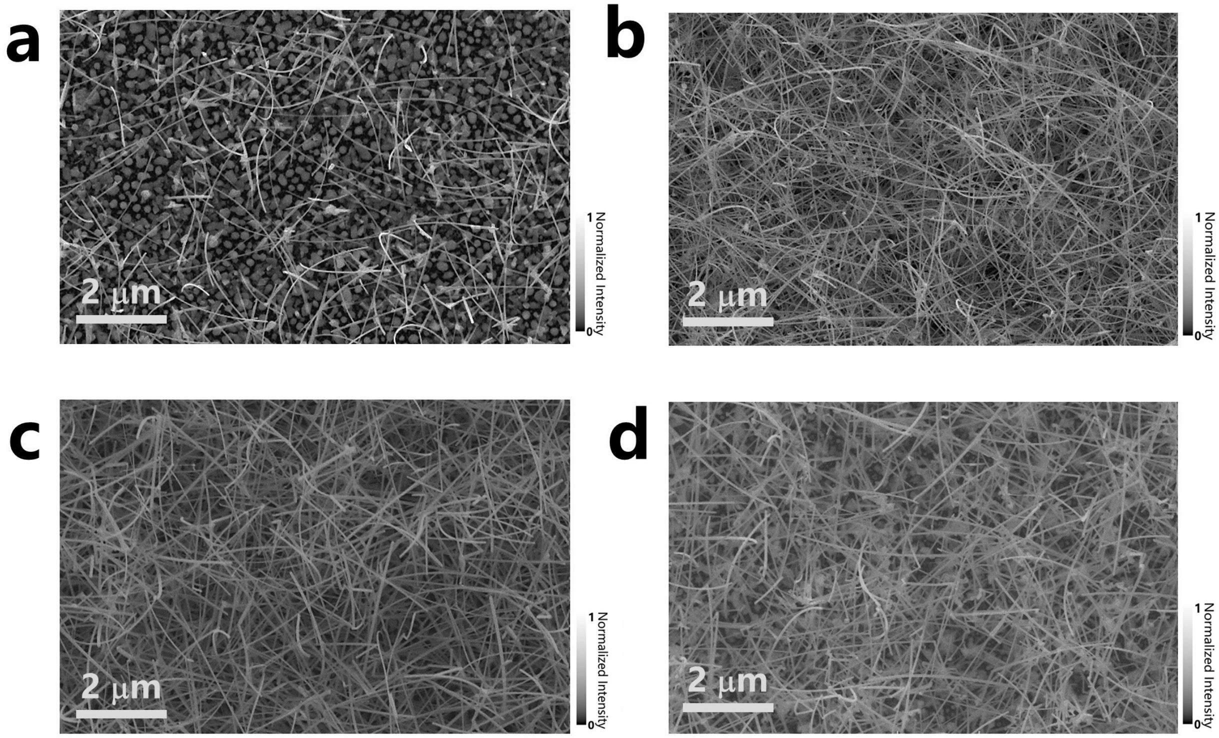

Supplementary Figure 2. Surface morphology of the obtained nanowires. Scanning electron microscope images of the as-prepared $In_xGa_{1-x}Sb$ nanowires. The weight ratio of InSb:GaSb is (a) 10:1, (b) 20:1, (c) 30:1. (d) NWs grown with a higher substrate temperature (510 ^oC) for the 30:1 weight ratio. The grey scales denote the measured intensity.

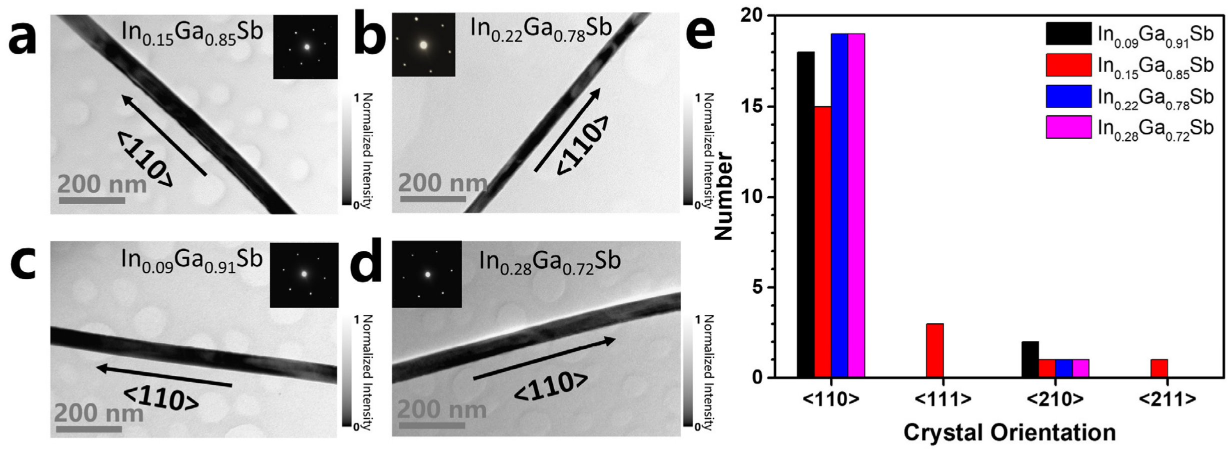

Supplementary Figure 3. Growth orientations of the as-prepared $In_xGa_{1-x}Sb$ NWs. (a-d) Transmission electron microscopy images of the representative $In_{0.15}Ga_{0.85}Sb$, $In_{0.22}Ga_{0.78}Sb$, $In_{0.09}Ga_{0.91}Sb$ and $In_{0.28}Ga_{0.72}Sb$ NWs, respectively. All nanowires are grown with the <110> direction. The insets show the corresponding selected area electron diffraction pattern of the nanowires. The grey scales denote the measured intensity. (e) Statistics of the NW growth direction with different indium concentrations.

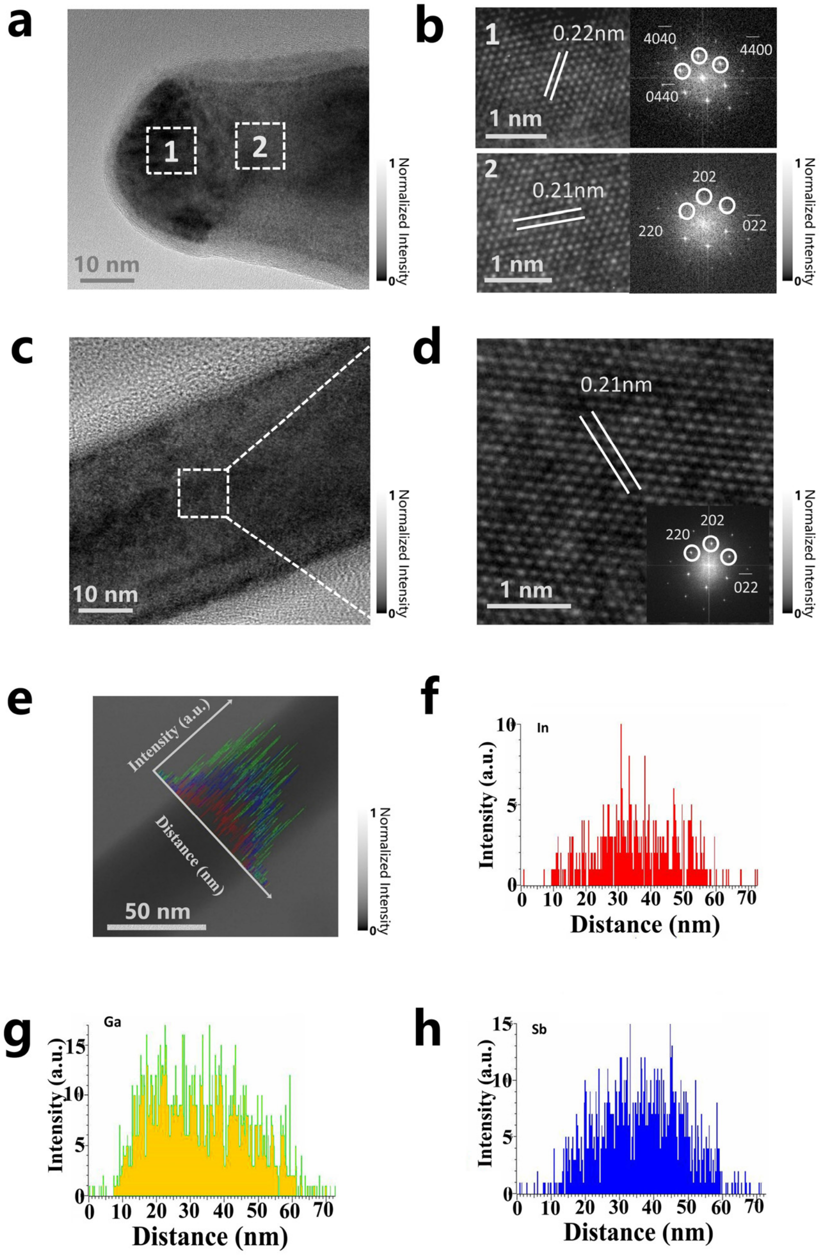

Supplementary Figure 4. Structure and composition of the typical $In_{0.09}Ga_{0.91}Sb$ nanowire. (a) High-resolution transmission electron microscopy (TEM) image of the tip and neck-bending region of the nanowire. (b) (1) Lattice fringes and (2) the corresponding fast Fourier transform (FFT) pattern of the tip and body regions of the nanowire, respectively. (c) High-resolution TEM image of the body region of the nanowire. (d) The corresponding lattice fringes marked in panel (c) with the inset showing its FFT pattern. (e) TEM image of the body region of the nanowire. Energy dispersive X-ray spectroscopy (EDS) line scan is performed on the marked location to assess the elemental composition. The grey scales denote the measured intensity. (f), (g) and (h) EDS line scan results of the element of In, Ga and Sb, respectively, along the radial direction of the nanowire. The measurement location is marked in panel (e).

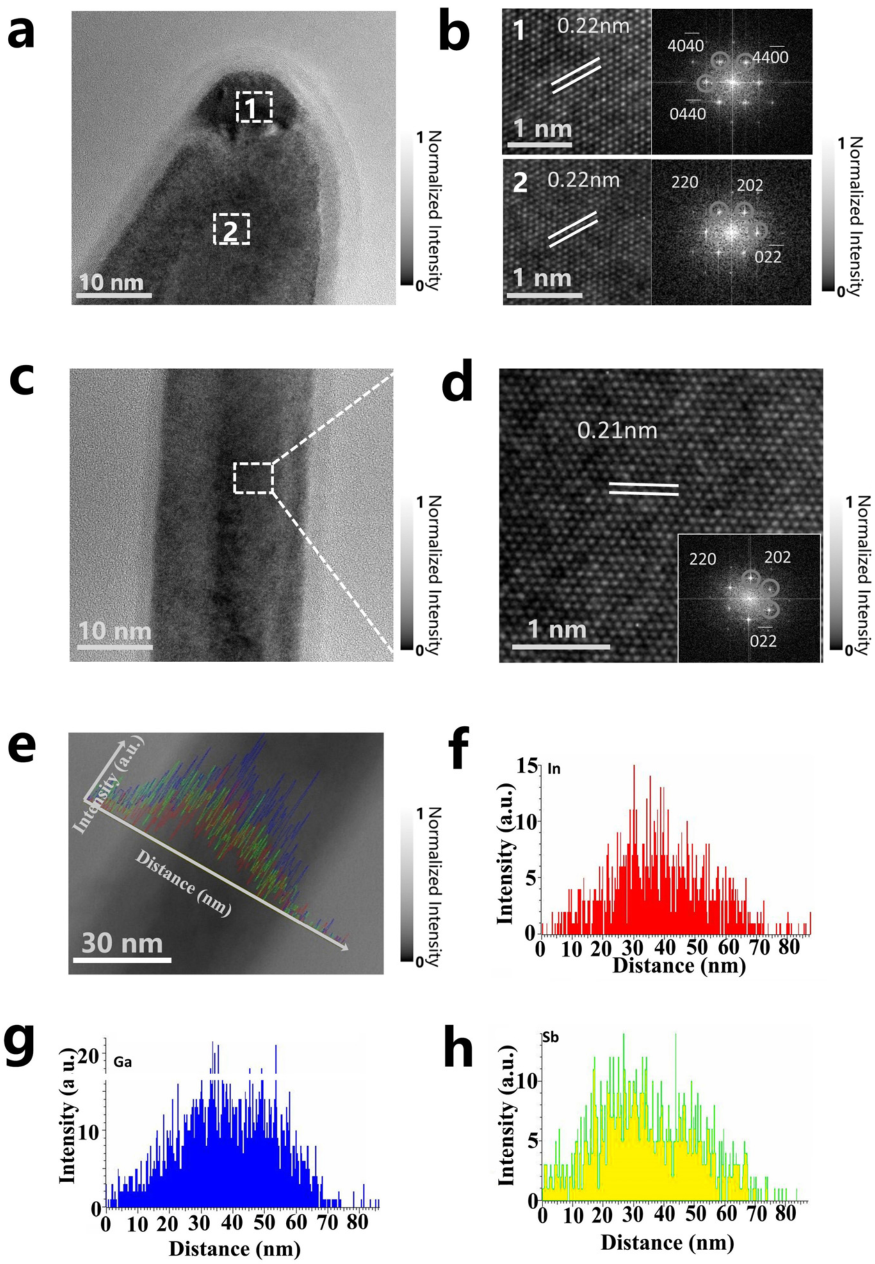

Supplementary Figure 5. Structure and composition of the typical $In_{0.15}Ga_{0.85}Sb$ nanowire. (a) High-resolution transmission electron microscopy (TEM) image of the tip and neck-bending region of the nanowire. (b) (1) Lattice fringes and (2) the corresponding fast Fourier transform (FFT) pattern of the tip and body regions of the nanowire, respectively. (c) High-resolution TEM image of the body region of the nanowire. (d) The corresponding lattice fringes marked in panel (c) with the inset showing its FFT pattern. (e) TEM image of the body region of the nanowire. Energy dispersive X-ray spectroscopy (EDS) line scan is performed on the marked location to assess the elemental composition. The grey scales denote the measured intensity. (f), (g) and (h) EDS line scan results of the element of In, Ga and Sb, respectively, along the radial direction of the nanowire. The measurement location is marked in panel (e).

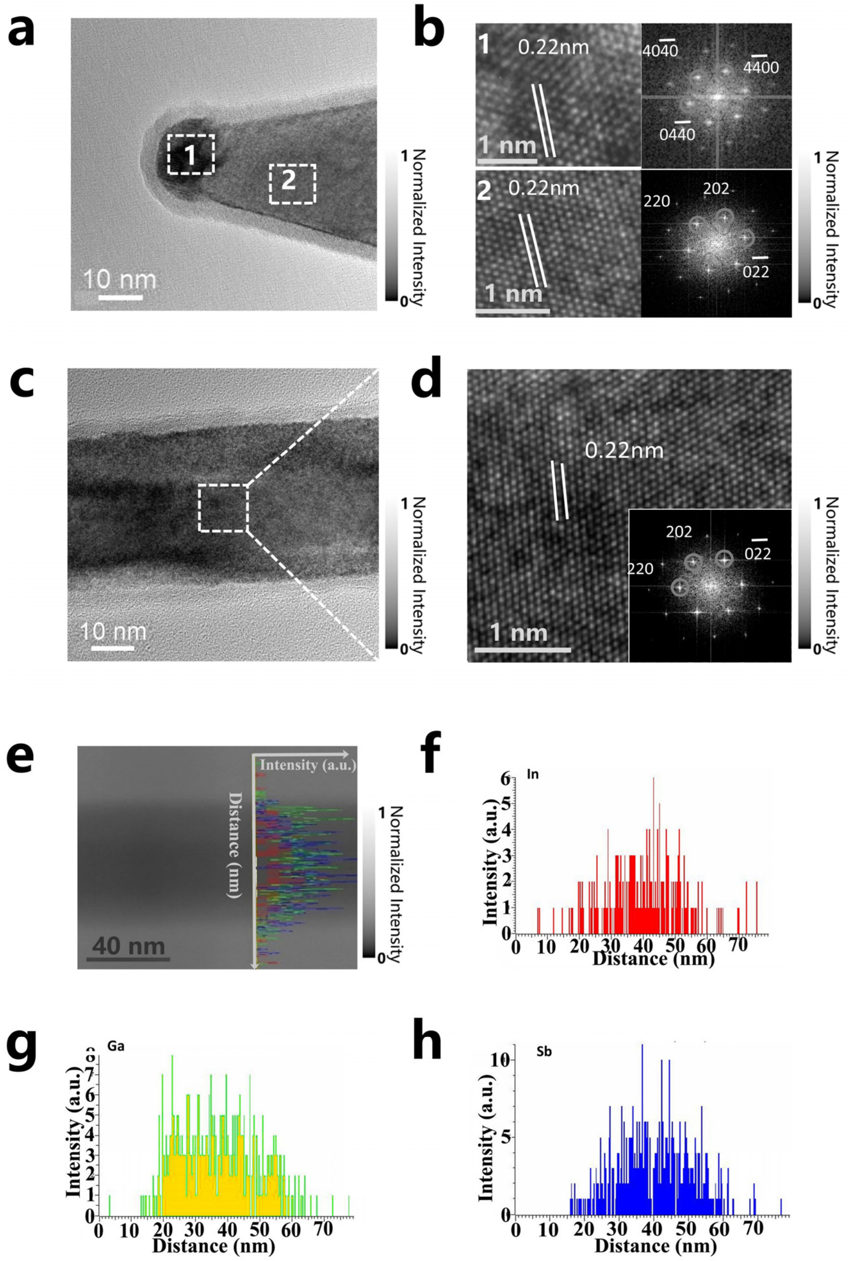

Supplementary Figure 6. Structure and composition of the typical $In_{0.22}Ga_{0.78}Sb$ nanowire. (a) High-resolution transmission electron microscopy (TEM) image of the tip and neck-bending region of the nanowire. (b) (1) Lattice fringes and (2) the corresponding fast Fourier transform (FFT) pattern of the tip and body regions of the nanowire, respectively. (c) High-resolution TEM image of the body region of the nanowire. (d) The corresponding lattice fringes marked in panel (c) with the inset showing its FFT pattern. (e) TEM image of the body region of the nanowire. Energy dispersive X-ray spectroscopy (EDS) line scan is performed on the marked location to assess the elemental composition. The grey scales denote the measured intensity. (f), (g) and (h) EDS line scan results of the element of In, Ga and Sb, respectively, along the radial direction of the nanowire. The measurement location is marked in panel (e).

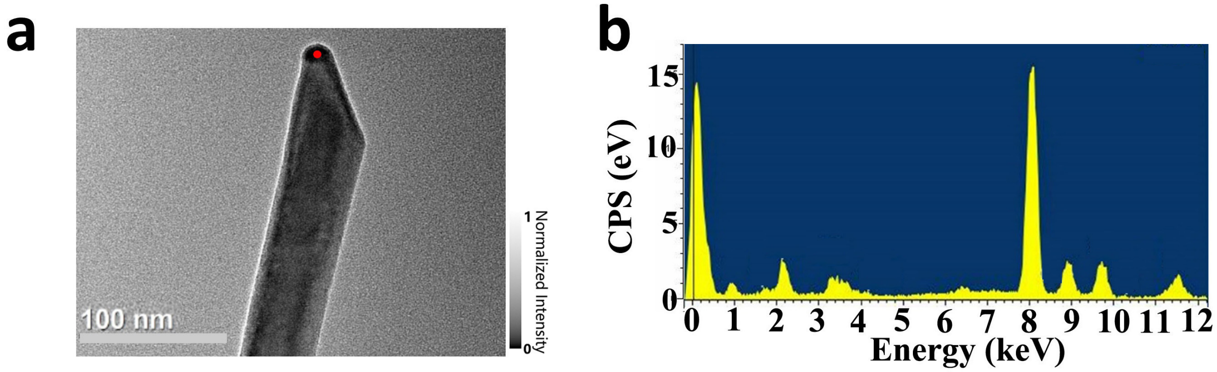

Supplementary Figure 7. Composition of the tip of a typical $In_{0.28}Ga_{0.72}Sb$ nanowire. (a) Transmission electron microscopy image of the nanowire. The grey scale denotes the measured intensity. (b) Energy dispersive X-ray spectroscopy results of the tip region of the nanowire. The measurement location is marked as a red dot in panel (a). The determined compositions are 74.6% Au and 25.4% In in atomic percentage.

Name and formula		Crystallographic parameters	
Reference code:	00-042-0821	Crystal system:	Hexagonal
PDF index name:	Gold Indium	Space group:	P63/m
Empirical formula:	Au10In3	Space group number:	176
Chemical formula:	Au10In3	a :	10.5387
		b :	10.5387
		c :	4.7862

No.	h	k	l	d [A]	2Theta[deg]	I [%]
1	1	1	0	5.27	16.81	20
2	0	2	0	4.56	19.451	20
3	0	1	1	4.245	20.91	20
4	1	1	1	3.542	25.122	50
5	1	2	0	3.449	25.811	30
6	0	2	1	3.305	26.956	50
7	0	3	0	3.04	29.356	5
8	0	3	1	2.57	34.882	5
9	1	3	0	2.532	35.423	80
10	0	0	2	2.396	37.507	80
**11**	**0**	**4**	**0**	**2.281**	**39.474**	**5**

Supplementary Table 3. The powder diffraction file (PDF) card of $Au_{10}In_3$. This crystal structure is consistent with the results obtained in Supplementary Figure 7b.

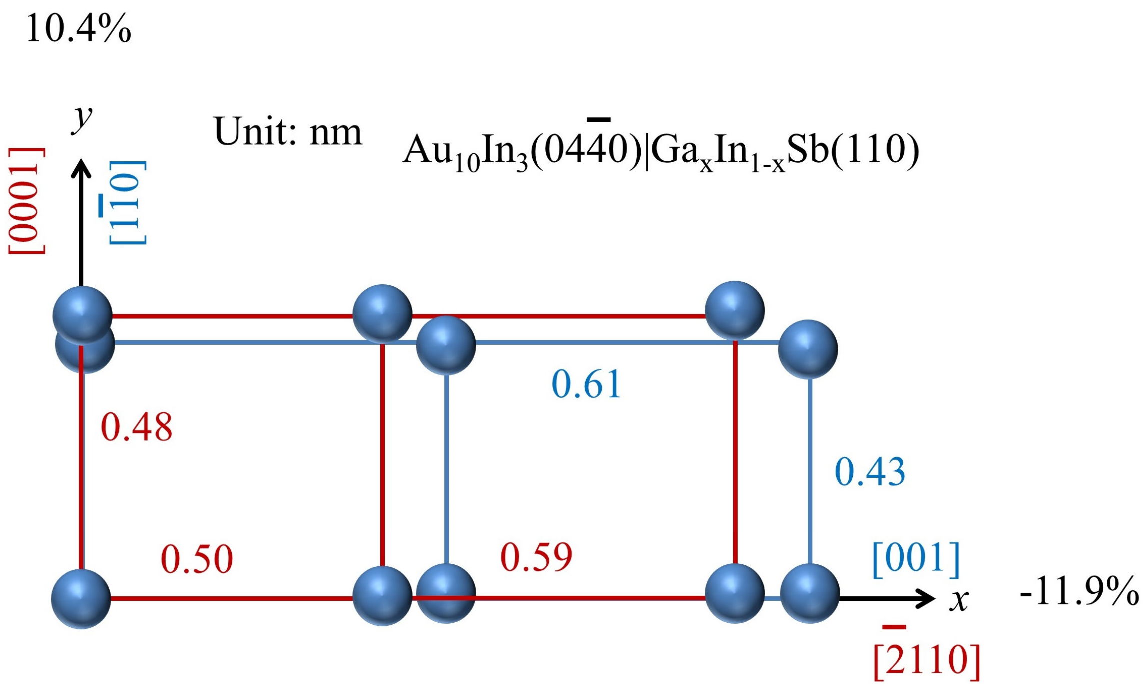

Supplementary Figure 8. In-plane orientation at the catalyst/nanowire interface. Schematic view of the in-plane orientation of In atom alignment in the catalyst/nanowires interface of $Au_{10}In_3(04\bar{4}0)|Ga_xIn_{1-x}Sb(110)$.

According to the previous study[1], the crystal structures and phases of the catalysts play the key role in growing high quality single crystalline NWs. To investigate the effect of Au-In alloy crystal phases on the growth behavior of InGaSb NWs, the interfacial In atom alignments between the catalytic seeds and NWs are simulated as shown in Supplementary Figure 8. Notably the In alignment in the $Au_{10}In_3$ $(04\bar{4}0)$ has the least lattice mismatch (~10% calculated as $(a_{catalyst} - a_{nanowire})/a_{catalyst}$ x100%) with the one in InGaSb (110), which would contribute efficiently to the <110> preferred growth orientation of the InGaSb NWs.

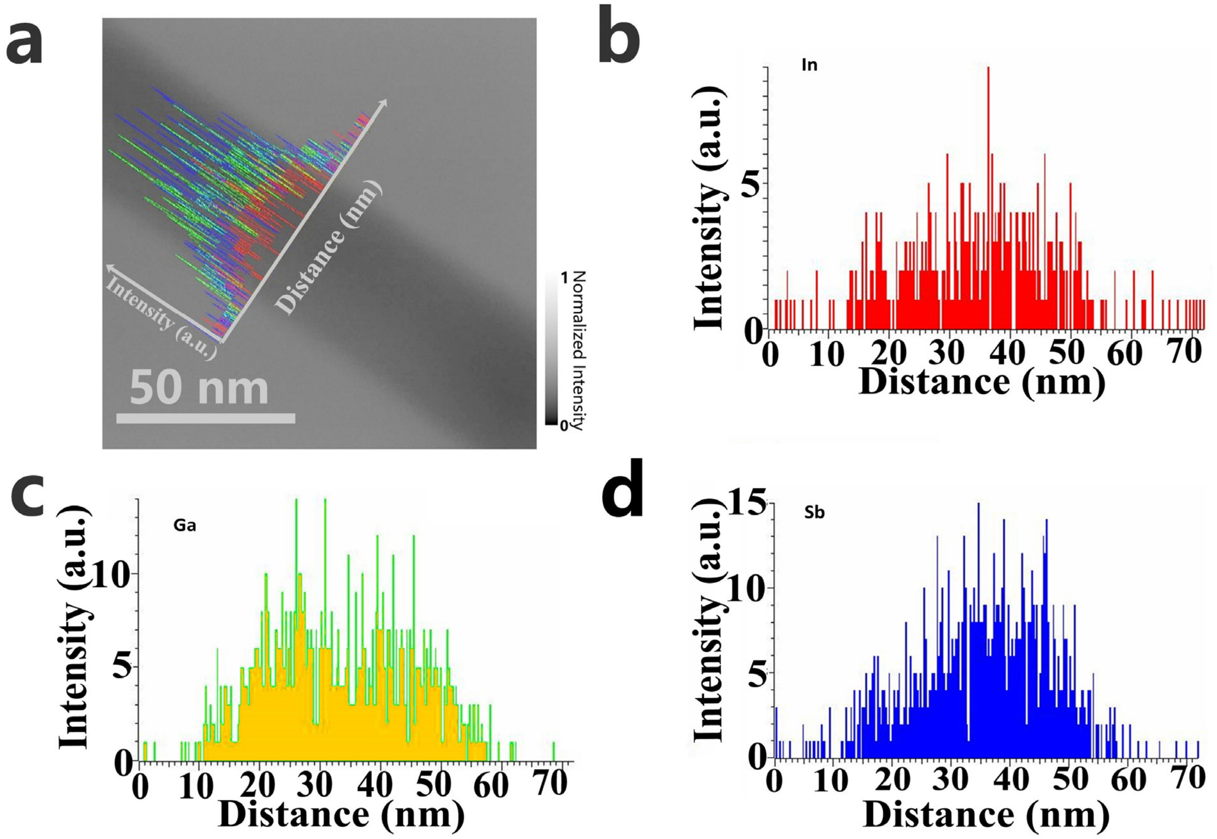

Supplementary Figure 9. Composition of the typical $In_{0.28}Ga_{0.72}Sb$ nanowire. (a) Transmission electron microscopy image of the nanowire. Energy dispersive X-ray spectroscopy (EDS) line scan is performed on the marked location to assess the elemental composition. The grey scale denote the measured intensity. (b-d) EDS line scan results of the element of In, Ga and Sb, respectively, along the radial direction of the nanowire. The measurement location is marked in panel (a).

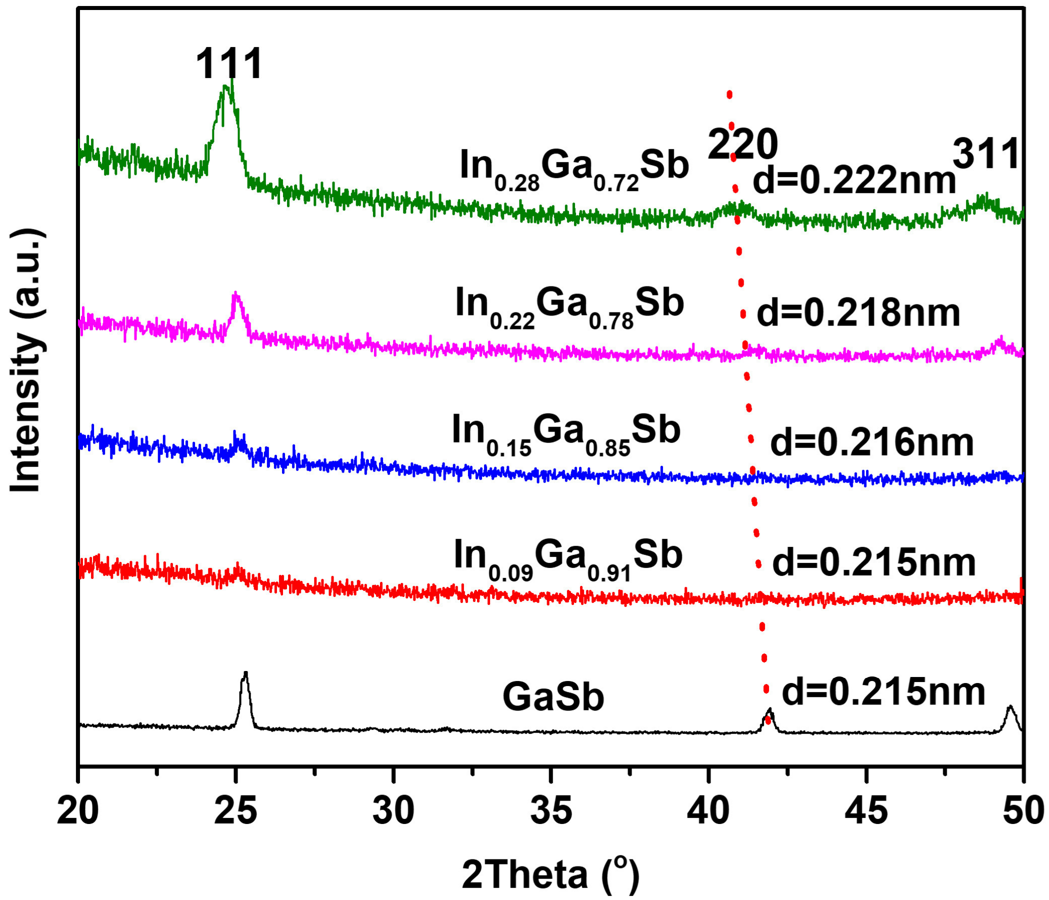

Supplementary Figure 10. X-ray diffraction (XRD) of the obtained $In_xGa_{1-x}Sb$ nanowires. Although transmission electron microscopy does not indicate the change of lattice spacing when the indium concentration changes, XRD results can clearly reveal the increase of lattice spacing when the indium concentration increases.

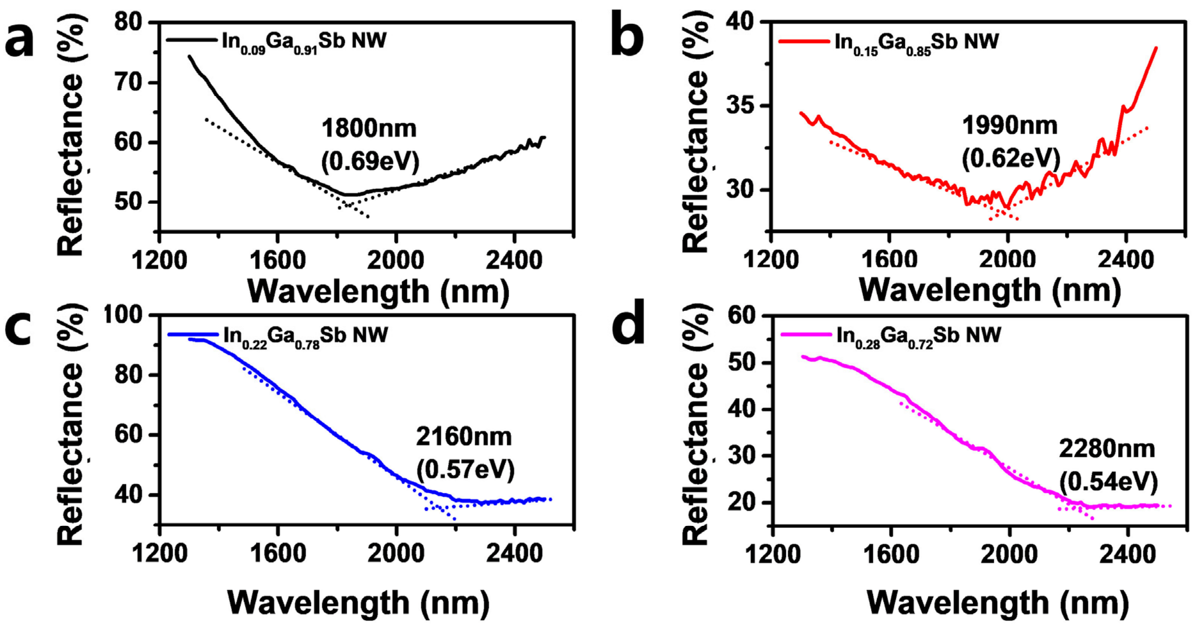

Supplementary Figure 11. Absorption spectra of the obtained $In_xGa_{1-x}Sb$ NWs. (a) $In_{0.09}Ga_{0.91}Sb$ nanowire. (b) $In_{0.15}Ga_{0.85}Sb$ nanowire. (c) $In_{0.22}Ga_{0.78}Sb$ nanowire. (d) $In_{0.28}Ga_{0.72}Sb$ nanowire.

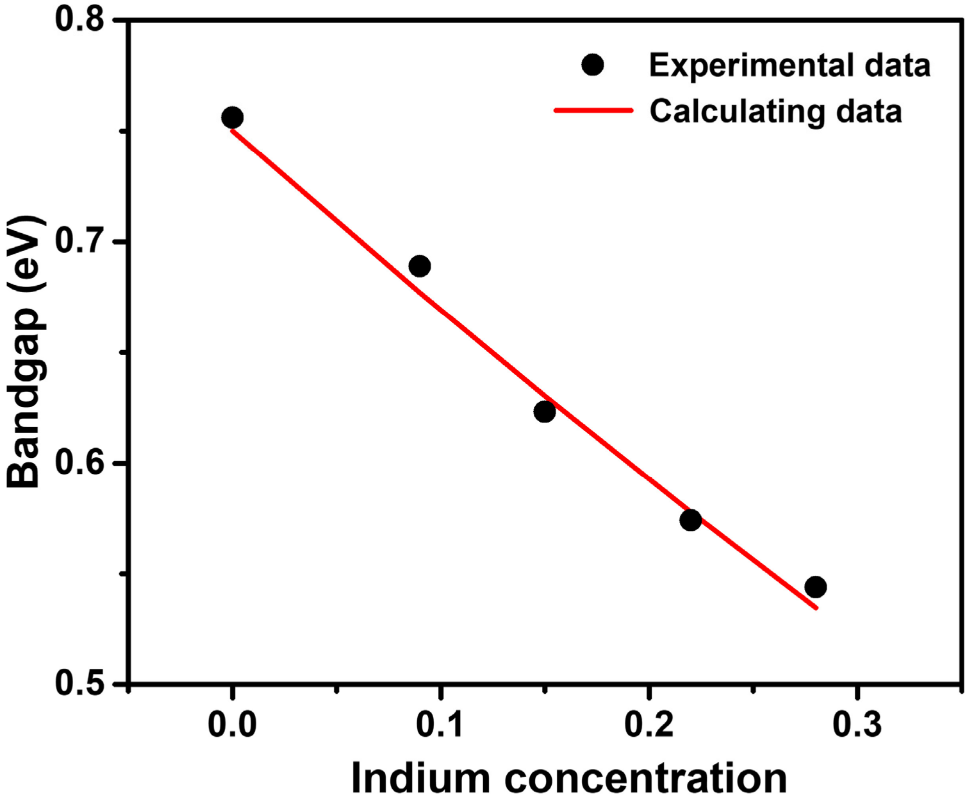

Supplementary Figure 12. Determined bandgap of the obtained $In_xGa_{1-x}Sb$ nanowires.

In order to assess the band gap of the $In_xGa_{1-x}Sb$ nanowires, reflective spectra of the nanowires were measured and shown in Supplementary Figure 11. The bandgap values of $In_xGa_{1-x}Sb$ can also be calculated from the following equation[2]:

$$E_g\,(In_xGa_{1-x}Sb) = E_g(GaSb)\,(1-x) + E_g(InSb)\,x - 0.22\,x(1-x) \qquad (1)$$

The calculated and measured band gap values as a function of indium concentration is shown in Supplementary Figure 12, which shows a good consistence between the measured and calculated values.

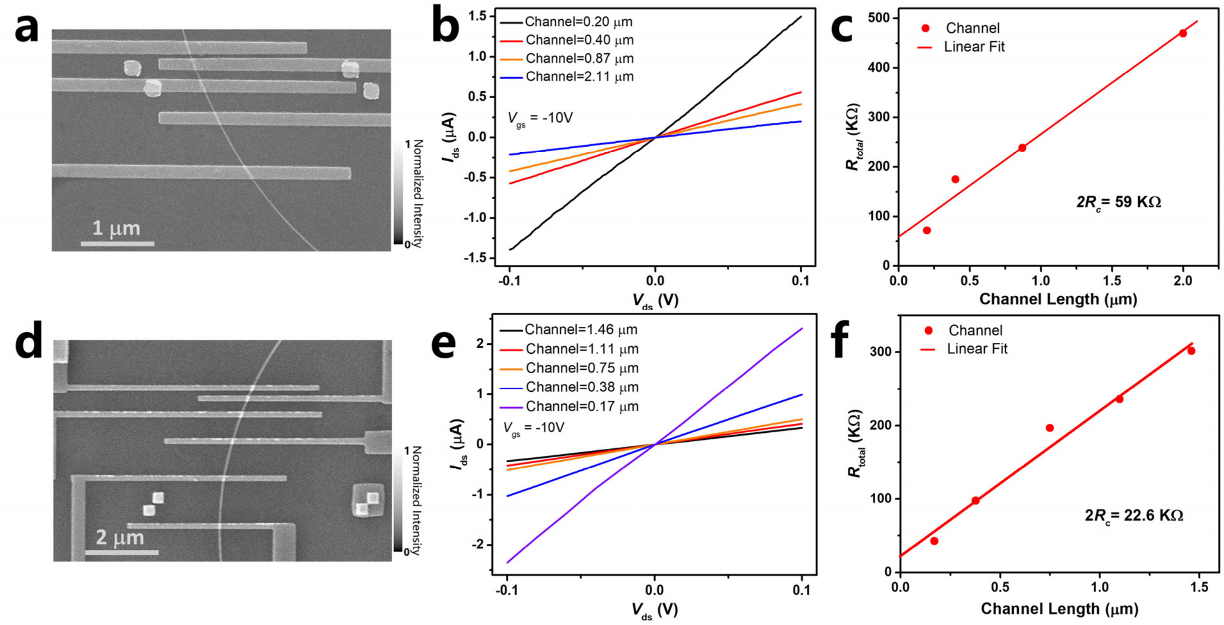

Supplementary Figure 13. Determination of the contact resistance of nanowire devices. (a) Scanning electron microscopy image of a nanowire device with multiple channel length fabricated on a SiO_2/Si substrate to extract the contact resistance using the transfer length method. (b) Current–Voltage curves of a single $In_{0.28}Ga_{0.72}Sb$ nanowire device. (c) Total resistance as a function of the channel length for the $In_{0.28}Ga_{0.72}Sb$ nanowire device with a gate bias of -10 V. The contact resistance is extracted to be 29.5 kΩ. (d, e, f) Repeat for the $In_{0.09}Ga_{0.91}Sb$ nanowire, in which the contact resistance is extracted to be 11.3 kΩ. The grey scales denote the measured intensity.

In order to determine the contact resistance of nanowire devices, transistors with multi-channel length were fabricated using a single nanowire as shown in Supplementary Figure 13a and 13d[3,4]. The device with the $In_{0.28}Ga_{0.72}Sb$ nanowire channel exhibits a contact resistance (R_c) of 29.5 kΩ, while the $In_{0.09}Ga_{0.91}Sb$ NW device yields a R_c of 11.3 kΩ. We can conclude that the contact resistance is only about 1/10 of the total resistance even at the device ON state, and this contact resistance would become orders of magnitude lower when the nanowire devices are turned into OFF state. Combined with the linear relationship between source-drain current and source-drain voltage, we can infer that the contact is Ohm-like here.

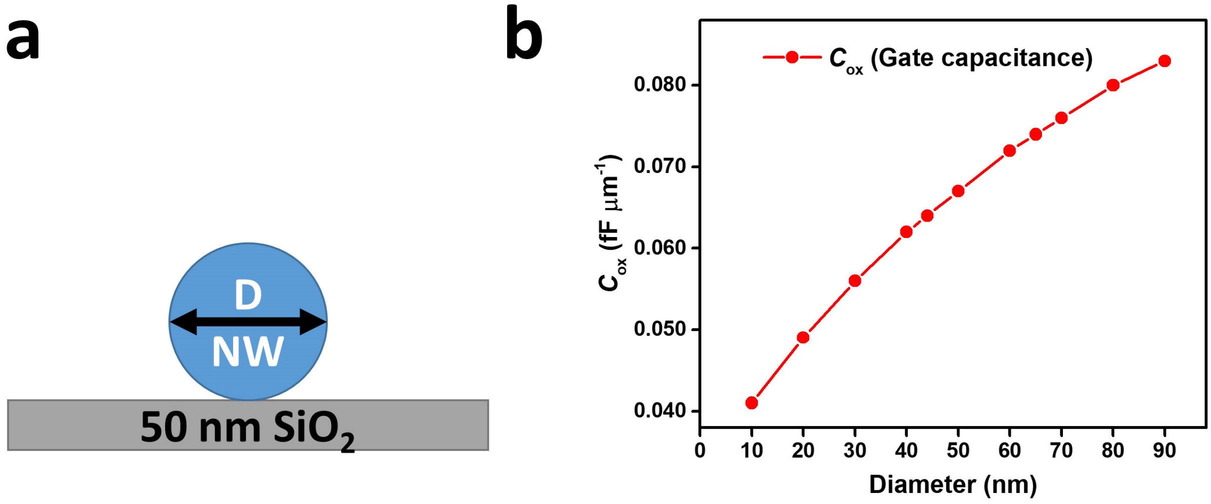

Supplementary Figure 14. Determination of the gate capacitance (C_{ox}) of nanowire devices. (a) Model for the calculation by using the COMSOL Multiphysics software. (b) Calculated C_{ox} per unit length as a function of the nanowire diameter.

Finite element method is used to calculate the gate capacitance of the nanowires by using COMSOL Multiphysics. The model for the calculation is shown in Supplementary Figure 14a. The calculated gate capacitance per unit length (c_{ox}) as a function of the nanowire diameter is also shown in Supplementary Figure 14b. Then, the gate capacitance of a predefined nanowire diameter can be calculated by the equation[5]:

$$C_{ox} = c_{ox}\, L \tag{2}$$

where L is the length of the nanowire.

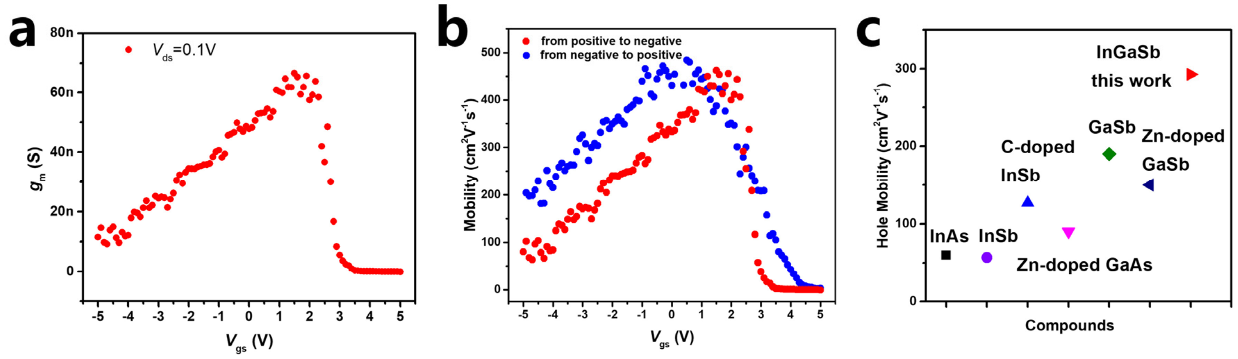

Supplementary Figure 15. Mobility calculation of a typical $In_{0.09}Ga_{0.91}Sb$ nanowire device. (a) The transconductance (g_m) as a function of gate voltage with a source-drain bias of 0.1 V. (b) The mobility calculation performed with the dual sweep measurement. It is confirmed that there is a neglectable error raised by the hysteresis associated with different sweep directions. (c) The average hole mobility of different representative III-V p-type semiconductor nanowires, including InAs, InSb, C-doped InSb, Zn-doped GaAs, GaSb, and Zn-doped GaSb[6-11].

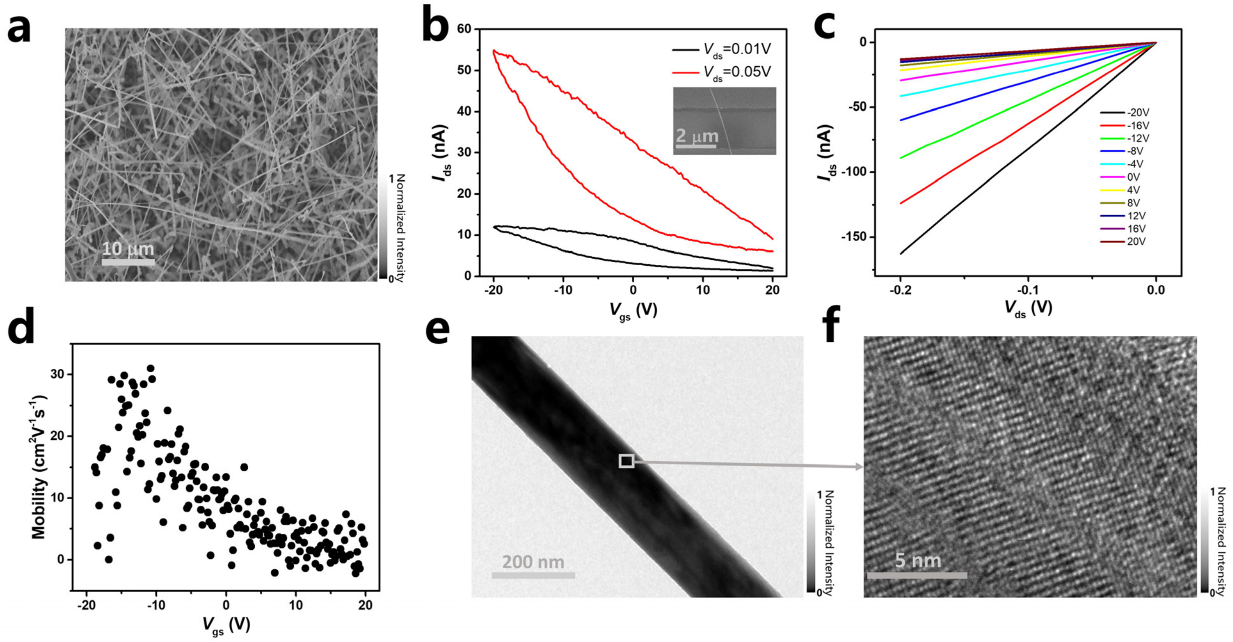

Supplementary Figure 16. Growth and characterization of GaSb nanowires. (a) Scanning electron microscopy (SEM) image of the obtained GaSb nanowires. (b) Transfer curves of a typical field-effect transistor based on a single GaSb nanowire. Inset shows the corresponding SEM image of the device. (c) Output curves of the same device presented in the panel (a). (d) Mobility as a function of gate bias with a source-drain bias of 0.01 V. (e) Transmission electron microscopy (TEM) image of a representative GaSb nanowire. (f) High-resolution TEM image. The grey scales denote the measured intensity.

In this work, GaSb nanowires (NWs) were also grown by the similar method as described in the main text. As depicted in the SEM image in Supplementary Figure 16a, it is clear that large quantities of NWs are grown on the substrate. The obtained GaSb NWs are long (> 30 µm), straight, dense and relatively thick in the diameter with an average value of 233 ± 60 nm. This relatively thick diameter is mainly due to the uncontrolled radial growth of GaSb NWs as reported in the literature[12]. In any case, as extracted from the electrical characterization (Supplementary Figures 16b and c), the calculated peak hole mobility of the typical single GaSb NW device is found to be only 33 cm^{2}V^{-1}s^{-1} (Supplementary Figure 16d) while the average hole mobility value of GaSb NW devices is 26 cm^{2}V^{-1}s^{-1} based on the statistics of 40 single NW devices. Also, based on the typical TEM and HRTEM images (Supplementary Figures 16e and f), it is obvious that there are many lattice defects (e.g. stacking faults and inversion domains, etc.) existed in the GaSb NW, which is in a distinct contrast to the ones of $In_xGa_{1-x}Sb$ NWs (Figure 2 and Supplementary Figures 4 to 6). These large amounts of defects could contribute to the relatively low mobility of GaSb NWs due to the severe carrier scattering there. Furthermore, these defects can also provide a large amount of free carriers that cause the down shift of the Fermi level of GaSb to its valance band maximum. As a result, it is difficult to deplete the free carriers of the NW to achieve the device OFF state by electrical back-gating.

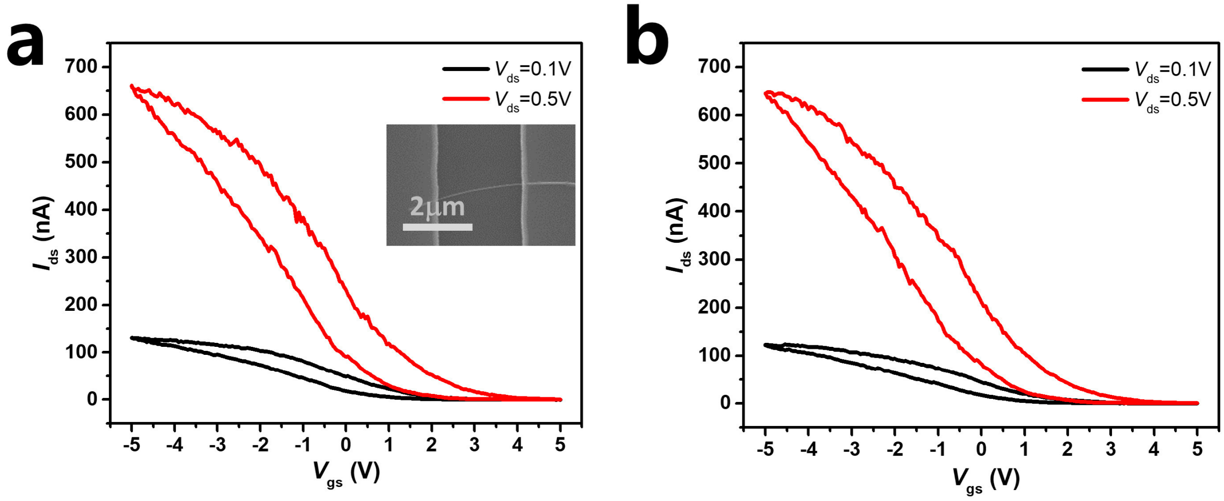

Supplementary Figure 17. Transfer curves of the typical $In_{0.09}Ga_{0.91}Sb$ nanowire device. (a) Measurement in air. Inset shows the scanning electron microscopy image of the corresponding nanowire device. (b) Measurement in vacuum (3.5×10^{-4} Pa).

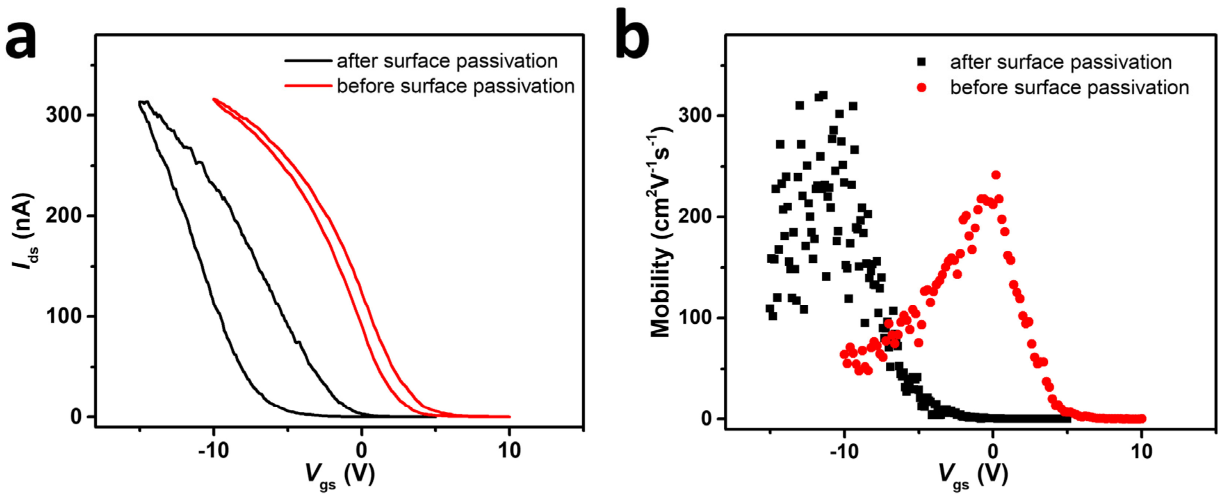

Supplementary Figure 18. Surface passivation of the $In_{0.09}Ga_{0.91}Sb$ nanowire device. (a) Transfer curves of a typical device measured before and after the passivation. (b) Mobility values extracted based on the transconductance of the device measured before and after the passivation. The gate voltage sweeping is conducted from the negative to positive bias.

In general, the mobility values of nanowire (NW) devices can be affected by the channel surface condition, such as the surface roughness, passivation and adsorbents, etc. In this work, since both GaSb and InGaSb NWs have the smooth surface as revealed from the transmission electron microscopy characterization (Figure 2, Supplementary Figures 4 to 6 and 16), the surface roughness effect should be the same among all these NWs. To further exclude the effect of any surface modification on the device mobility of the NWs, we measured the transfer curves of a typical $In_{0.09}Ga_{0.91}Sb$ nanowire transistor in both air and vacuum (3.5×10^{-4} Pa) as shown in Supplementary Figure 17. Explicitly, there is not any noticeable difference observed for the measurement result in both air and vacuum, which suggests that the adsorbents have insignificant effect on the electrical properties of NW devices. Then, surface passivation with ammonia sulfide $(NH_4)_2S$ were also performed on the $In_{0.09}Ga_{0.91}Sb$ nanowire transistor. The fabricated devices were first soaked in $(NH_4)_2S$ solution (stock solution) for 40 s. After that, the devices were rinsed carefully using deionized water and ethanol, and then baked at 120 °C for ~5 h[13]. As presented in Supplementary Figure 18, the surface passivation can slightly reduce the subthreshold swing and move the subthreshold voltage towards the negative voltage direction by minimizing the surface trap concentration on the nanowire device channel, which can further enhance the corresponding device mobility to some extent[13]. This phenomenon indicates that the initial high device mobility values of our $In_{0.09}Ga_{0.91}Sb$ nanowires are indeed attributed to their intrinsic material properties, instead of relating to any surface passivation effect. In this case, the effective electrical back-gating to efficiently turning the device ON and OFF states can also be attributed to the intrinsic nanowire properties, rather than any other extrinsic effect (e.g. surface modification).

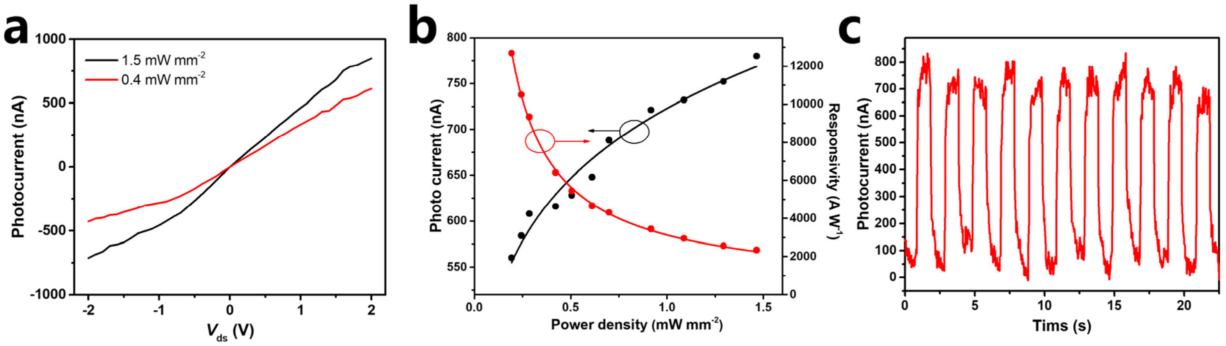

Supplementary Figure 19. Photodetection of the $In_{0.28}Ga_{0.72}Sb$ nanowire (405 nm laser). (a) Photocurrent versus drain-source bias under different light intensity illumination. (b) Photocurrent and responsivity as a function of the light intensity. (c) Transient response under modulated light illumination with intensity of 1.5 mW mm^{-2}. The measurement is performed with a source-drain bias of 2 V and a gate bias of 0 V.

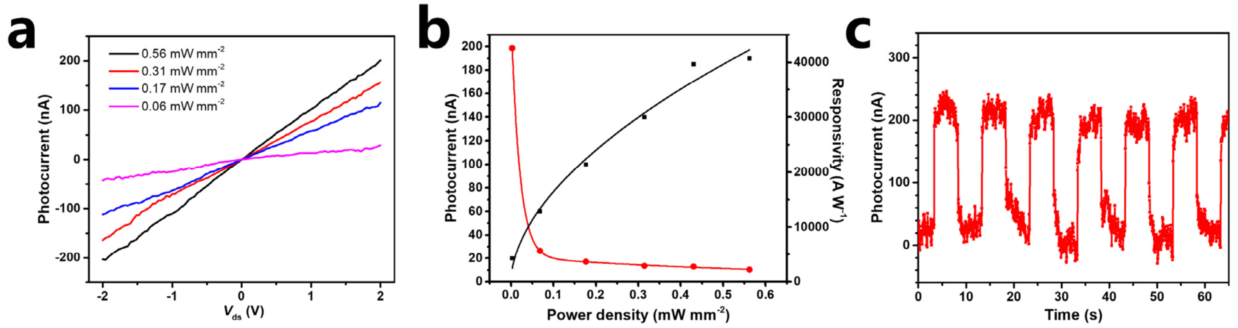

Supplementary Figure 20. Photodetection of the $In_{0.28}Ga_{0.72}Sb$ nanowire (635 nm laser). (a) Photocurrent of the nanowire device under different illumination intensities. (b) Photocurrent and responsivity as a function of the different incident light intensity. By nonlinear fitting of $I_{ph} = A\,\Phi^{\alpha}$, the α value is founded to be 0.55, where I_{ph} is the photocurrent and Φ is the light intensity. (c) Photoresponse measurement of the nanowire device under the illumination intensity of 0.5 mW mm^{-2}. The measurement is performed with a source-drain bias of 2 V and a gate bias of 0 V.

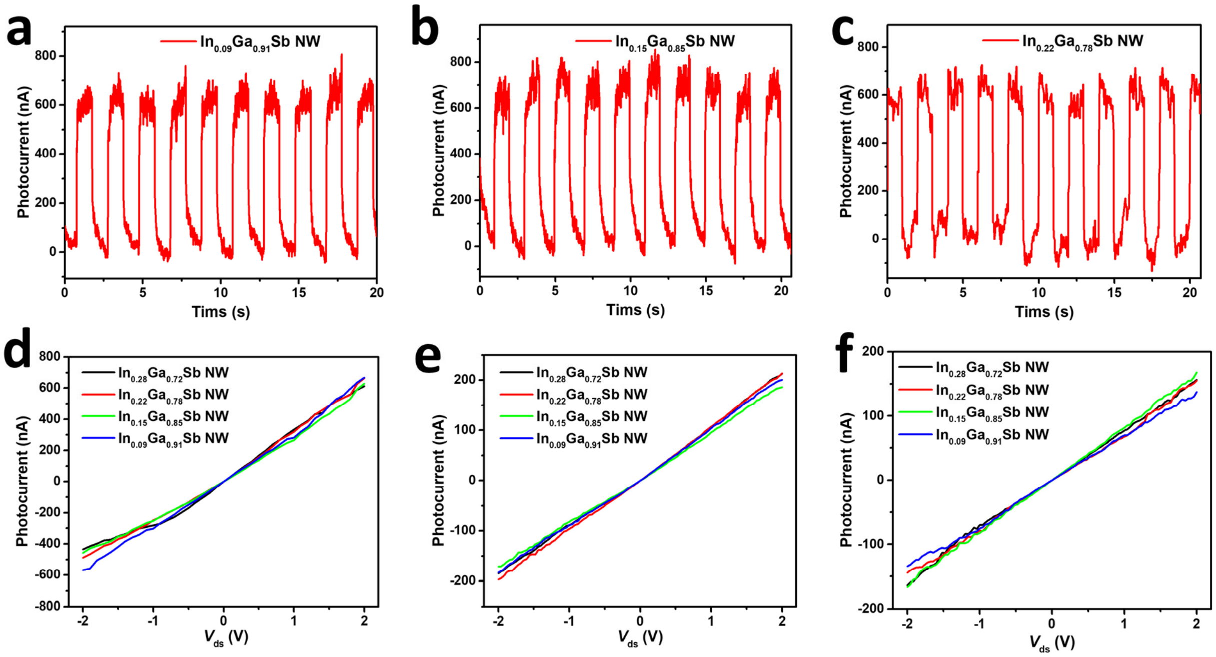

Supplementary Figure 21. Photoresponse of the single $In_xGa_{1-x}Sb$ nanowire. (a) Transient performance of the $In_{0.09}Ga_{0.91}Sb$ nanowire photodetector. (b) Transient performance of the $In_{0.15}Ga_{0.85}Sb$ nanowire photodetector. (c) Transient performance of the $In_{0.22}Ga_{0.78}Sb$ nanowire photodetector. The measurements in panel (a) to (c) are performed under an illumination of 405 nm with an intensity of 0.5 mW mm^{-2}, a source-drain bias of 2 V and a gate bias of 0 V. Photodetection performance of the single nanowire devices with different nanowire composition and illumination conditions. (d) 405 nm, 0.4 mW mm^{-2}. (e) 532 nm, 1.6 mW mm^{-2}. (f) 635nm, 0.3 mW mm^{-2}. The diameter of the NWs is 50 nm ± 5 nm for all the measurements.

**InGaSb Photodetector (405nm)**	**Responsivity (AW^{-1})**	**EQE (%)**	**Detectivity (Jones)**	**Response Time (rise time)(μs)**	**Response Time (decay time) (μs)**
**$In_{0.09}Ga_{0.91}Sb$**	2405	$7.37x10^6$	$1.78x10^9$	42	53
**$In_{0.15}Ga_{0.85}Sb$**	2293	$7.02x10^6$	$1.71x10^9$	36	51
**$In_{0.22}Ga_{0.78}Sb$**	2459	$7.53x10^6$	$1.81x10^9$	32	43
**$In_{0.28}Ga_{0.72}Sb$**	2332	$7.14x10^6$	$1.80x10^9$	45	58

Supplementary Table 4. The photodetection performance of $In_xGa_{1-x}Sb$ nanowire devices (405 nm, 0.4 mW mm^{-2}). EQE stands for external quantum efficiency.

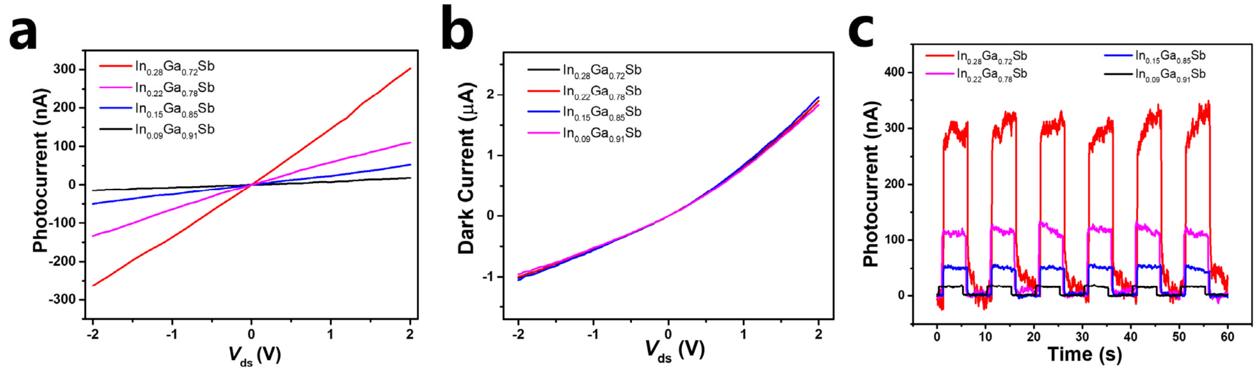

Supplementary Figure 22. Photodetection of the single $In_xGa_{1-x}Sb$ nanowire (1550 nm laser). (a) Current–Voltage curves of $In_xGa_{1-x}Sb$ nanowire devices. (b) The dark current of $In_xGa_{1-x}Sb$ nanowire devices. (c) Transient response of the single $In_xGa_{1-x}Sb$ NW photodetector devices. The illumination intensity is 3.1 mW mm^{-2} for all the measurements.

InGaSb Photodetector (1550nm)	Responsivity (AW^{-1})	EQE (%)	Detectivity (Jones)	Response Time (rise time)(μs)	Response Time (decay time) (μs)
$In_{0.09}Ga_{0.91}Sb$	50	$4.0x10^4$	$3.08x10^7$	32	41
$In_{0.15}Ga_{0.85}Sb$	117	$9.4x10^4$	$7.18x10^7$	45	68
$In_{0.22}Ga_{0.78}Sb$	209	$1.67x10^5$	$1.28x10^8$	37	49
$In_{0.28}Ga_{0.72}Sb$	434	$3.48x10^5$	$2.66x10^8$	38	53

Supplementary Table 5. The photodetection performance of $In_xGa_{1-x}Sb$ nanowires (1550 nm, 3.1 mW mm^{-2}). EQE stands for external quantum efficiency.

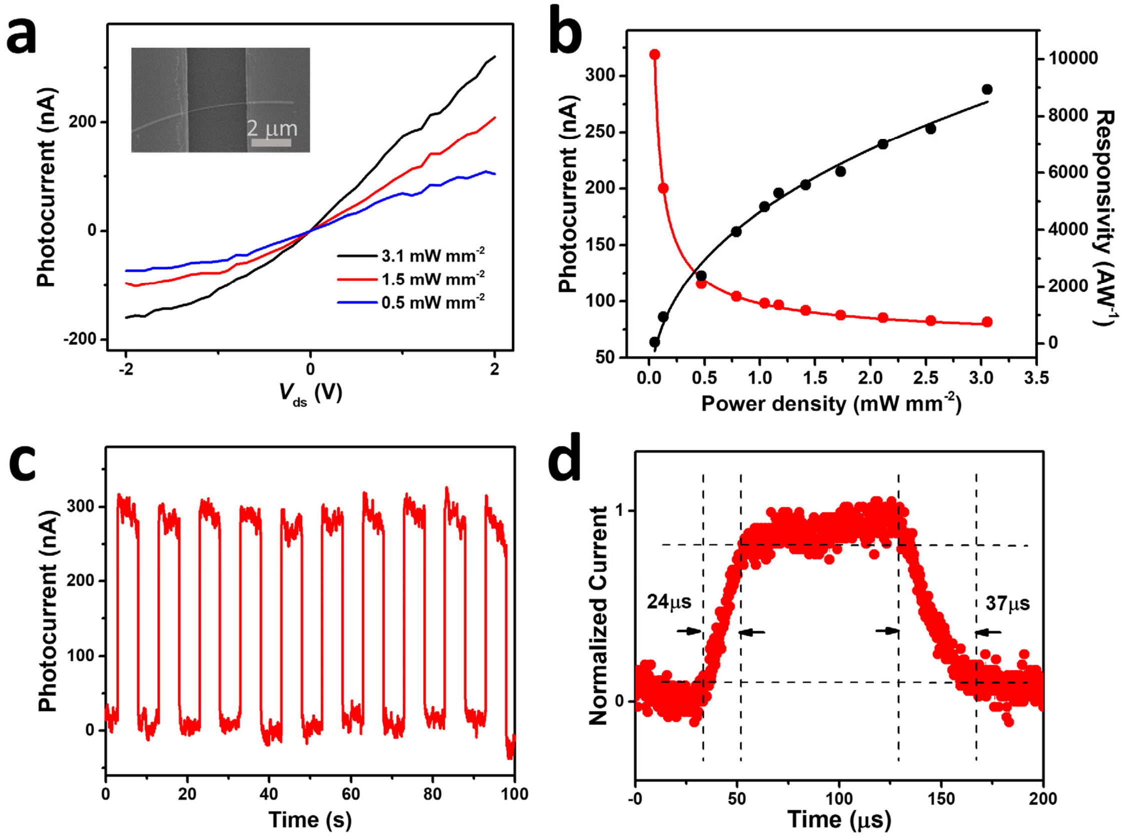

Supplementary Figure 23. Photodetection of the nanowire device after channel passivation. (a) Current–Voltage curves under the illumination of different light intensity. Inset shows the scanning electron microscopy image of the passivated nanowire device. (b) Photocurrent and responsivity as a function of the incident illumination intensity. (c) Transient response of the photodetector. The light intensity is 3.1 mW mm^{-2} with a chopped frequency of 0.1 Hz. (d) High-resolution transient curve of the device. For all measurements, a single $In_{0.28}Ga_{0.72}Sb$ nanowire with surface passivation of $(NH_4)_2S$ is employed as the device channel, while a source-drain bias of 2 V, a gate bias of 0 V and an illumination of 1550 nm are utilized.

For surface passivation, the fabricated devices were soaked in $(NH_4)_2S$ solution (stock solution) for 40 s. After that, the devices were rinsed carefully using deionized water and ethanol, and then baked at 120 °C for ~5 h[13]. The photodetection performance of the $(NH_4)_2S$ passivated $In_{0.28}Ga_{0.72}Sb$ nanowire (NW) photodetector device is then presented in Supplementary Figure 23. It is clear that the rise and decay time constants of the passivated InGaSb NW device are reduced from 38 and 53 µs (without passivation) to 24 and 37 µs (with passivation), accordingly. These reduced response times can be explained by the minimized surface trap concentration owing to the effective surface passivation. Since the difference of the observed response times is relatively small as compared between with and without the surface passivation, all the discussion presented in this work would be based on the results without any surface passivation. In any case, the optical response can be confirmed due to the intrinsic properties of our InGaSb NWs, instead of the material quality issue.

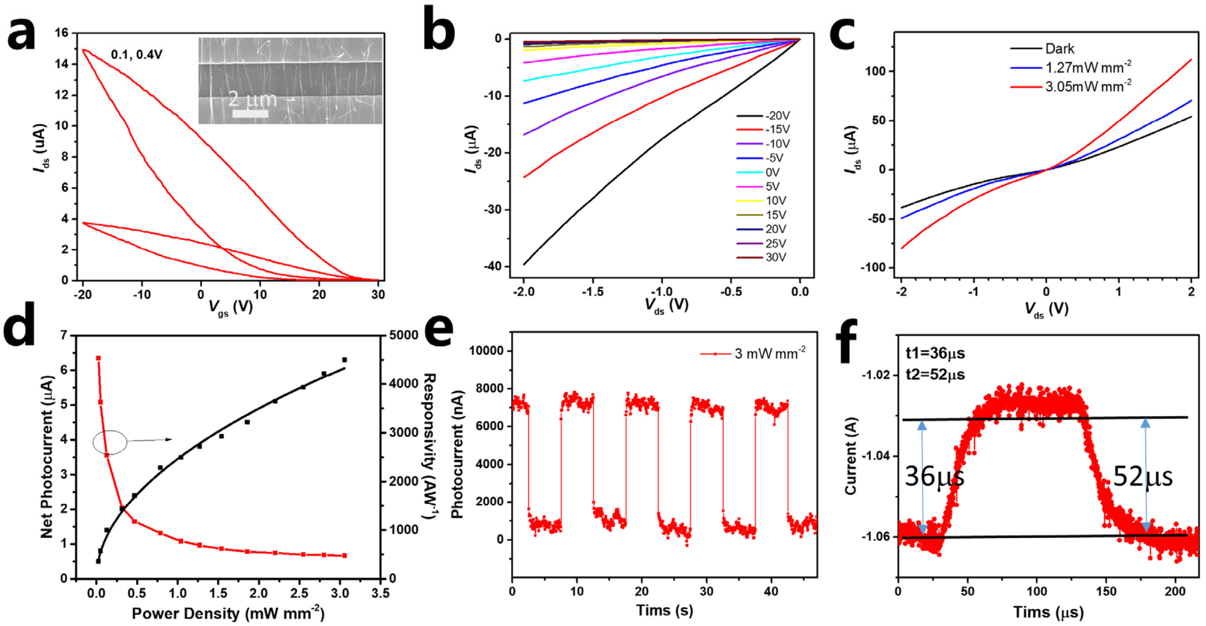

Supplementary Figure 24. $In_{0.28}Ga_{0.72}Sb$ nanowire parallel array devices. (a) Transfer curves of the device. Inset shows the corresponding scanning electron microscopy image of the device. (b) Output curves of the device. (c) Photocurrent versus drain-source bias of the device under different light intensities. (d) Photocurrent and photoresponsivity of the device as function of the light intensity. (e) Transient curve of the device with modulated illumination (1550 nm, 3 mW mm^{-2}). (f) High-resolution transient curve of the device with modulated illumination (1550 nm, 3 mW mm^{-2}).

Supplementary References

(1) Han N.; Wang Y.; Yang Z.-x.; Yip S.; Wang Z.; Li D.; Hung T. F.; Wang F.; Chen Y.; Ho J. C. Controllable III-V nanowire growth via catalyst epitaxy. *Journal of Materials Chemistry C.* **5**, 4393-4399, (2017).

(2) Vurgaftman I.; Meyer J. R.; Ram-Mohan L. R. Band parameters for III–V compound semiconductors and their alloys. *Journal of Applied Physics*, **89**, 5815-5875, (2001)

(3) Guo L. W.; Lu W.; Bennett B. R.; Boos J. B.; Alamo J. A. d. Ultralow Resistance Ohmic Contacts for p-Channel InGaSb Field-Effect Transistors. *IEEE Electron Device Letters*, **36**, 546-548, (2015)

(4) Berger H. H. Contact Resistance and Contact Resistivity. *Journal of The Electrochemical Society*, **119**, 507-514, (1972)

(5) Ford A. C.; Ho J. C.; Chueh Y. L.; Tseng Y. C.; Fan Z. Y.; Guo J.; Bokor J.; Javey A. Diameter-Dependent Electron Mobility of InAs Nanowires. *Nano Letters*, **9**, 360-365, (2009)

(6) Ford, A. C.; Chuang, S.; Ho, J. C.; Chueh, Y.-L.; Fan, Z.; Javey, A., Patterned p-doping of InAs nanowires by gas-phase surface diffusion of Zn. *Nano Lett.* 2010**,** *10*, 509-513.

(7) Khan, M. I.; Penchev, M.; Jing, X.; Wang, X.; Bozhilov, K. N.; Ozkan, M.; Ozkan, C. S., Electrochemical growth of InSb nanowires and report of a single nanowire field effect transistor. *Journal of Nanoelectronics and Optoelectronics*, **3**, 199-202, (2008)

(8) Yang, Z.-x.; Han, N.; Wang, F.; Cheung, H.-Y.; Shi, X.; Yip, S.; Hung, T.; Lee, M. H.; Wong, C.-Y.; Ho, J. C., Carbon doping of InSb nanowires for high-performance p-channel field-effect-transistors. *Nanoscale*, **5**, 9671-9676, (2013)

(9) Gutsche, C.; Regolin, I.; Blekker, K.; Lysov, A.; Prost, W.; Tegude, F. J., Controllable p-type doping of GaAs nanowires during vapor-liquid-solid growth. *Journal of Applied Physics*, **105**, 024305, (2009)

(10) Yang, Z.-x.; Yip, S.; Li, D.; Han, N.; Dong, G.; Liang, X.; Shu, L.; Hung, T. F.; Mo, X.; Ho, J. C., Approaching the Hole Mobility Limit of GaSb Nanowires. *ACS Nano*, **9**, 9268-9275, (2015).

(11) Babadi, A. S.; Svensson, J.; Lind, E.; Wernersson, L.-E., Impact of doping and diameter on the electrical properties of GaSb nanowires. *Applied Physics Letters* ,**110**, 053502, (2017).

(12) Yang, Z.-x.; Han, N.; Fang, M.; Lin, H.; Cheung, H.-Y.; Yip, S.; Wang, E.-J.; Hung, T.; Wong, C.-Y.; Ho, J. C., Surfactant-assisted chemical vapour deposition of high-performance small-diameter GaSb nanowires. *Nature Communications*, **5**, 5249, (2014).

(13) Cheung, H.Y.; Yip, S.P.; Han, N.; Dong, G.; Fang, M.; Yang, Z.; Wang, F.Y.; Lin, H.; Wong, C.Y.; Ho, J.C., Modulating Electrical Properties of InAs Nanowires via Molecular Monolayers. *ACS Nano*, **9**, 7545-7552, (2015).
